# VEXAS-Syndrom

**DOI:** 10.1007/s00393-024-01577-w

**Published:** 2024-09-28

**Authors:** Martin Krusche

**Affiliations:** Sektion für Rheumatologie und Entzündliche Systemerkrankungen in der III. Medizin, Universitätsklinikum Hamburg-Eppendorf (UKE), Martinistr. 52, 20246 Hamburg, Deutschland

Im Jahr 2020 wurde erstmals von David Beck et al. im *New England Journal of Medicine* eine neue autoinflammatorische Erkrankung mit dem Namen VEXAS-Syndrom beschrieben [1]. Das Akronym VEXAS steht für *V*acuoles, *E*1 enzyme, *X*-linked, *A*utoinflammatory, *S*omatic. Pathophysiologisch kommt es bei der Erkrankung durch eine somatisch erworbene Mutation des *UBA1*-Gens zu einer Fehlfunktion des Proteasoms mit konsekutiver Hochregulation multipler proinflammatorischer Zytokine (IL‑1, IL‑6, TNF-alpha, NFkB) in den erythrozytären und myeloiden Vorläuferzellen im Knochenmark.

Gekennzeichnet ist die Erkrankung durch einen (hyper)inflammatorischen Phänotyp, der eine Plethora an klinischen Krankheitssymptomen hervorrufen kann. Die Erkrankung kann sich als Mimik verschiedenster rheumatologischer Erkrankungen, wie z. B. der rezidivierenden Polychondritis, Riesenzellarteriitis oder Panarteriitis nodosa, manifestieren, aber weist auch häufig hämatologische Auffälligkeiten (v. a. ein myelodysplastisches Syndrom [MDS] in bis zu 50 %) und/oder dermatologische Veränderungen (z. B. neutrophile Dermatitis [vgl. Abb. 1]) auf.

**Abb. 1:**
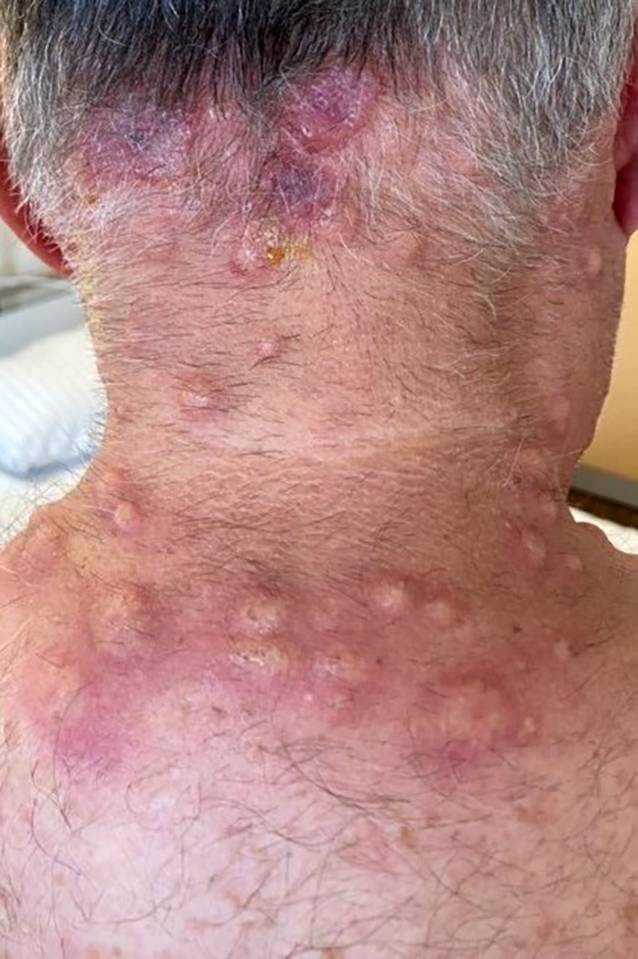
74-jähriger Patient mit ausgeprägter steroidrefraktärer neutrophiler Dermatitis bei UBA1-Mutation, der initial als Sweet-Syndrom behandelt wurde

Aufgrund des höheren Erkrankungsalters, der hyperinflammatorischen (oft multisystemischen) Krankheitsmanifestationen, der Komorbiditäten der Erkrankten sowie der Progressionstendenz ist die Behandlung des VEXAS-Syndroms oft eine Herausforderung.

Aktuell liegen aufgrund der Neuartigkeit der Erkrankung noch keine allgemeinen Therapiealgorithmen oder Krankheitsaktivitätsscores vor. Erkenntnisse und Empfehlungen bezüglich der Therapie beruhen überwiegend auf retrospektiven Analysen und kleineren Fallserien. Aktuell gibt es keine randomisiert kontrollierten Studien zur Therapie der Erkrankung, sodass das Evidenzlevel der hier vorgestellten Therapieoption noch sehr gering ist.

Die 2 wesentlichen Therapieprinzipien in der Behandlung des VEXAS-Syndroms bestehen aktuell 1) in der Kontrolle der (Hyper‑)Inflammation durch Immunsuppressiva und 2) in der Adressierung des pathologischen Knochenmarkklons.

Darüber hinaus ist die Mitbehandlung der Komorbiditäten (z. B. Transfusionsbedürftigkeit, Infektionsprophylaxe sowie Reduktion des Thromboembolierisikos) wichtig [2].

Bezüglich der Kontrolle der (Hyper‑)Inflammation mittels Immunsuppressiva hat sich bisher gezeigt, dass ein Großteil der Patienten einen hohen Steroidbedarf hat und sich häufig im Krankheitsverlauf eine zunehmende Steroidtoleranz entwickelt.

Die French VEXAS Group veröffentlichte im Jahr 2024 die bisher größte retrospektive Analyse bezüglich steroidsparender Therapieansätze [3]: Hierfür wurden 110 Patienten, die 194 zielgerichtete Therapien erhielten, analysiert. Hiervon erhielten 40 % JAK-Inhibitoren (JAKi), 26 % IL-6-Inhibitoren (IL-6i), 17 % IL-1-Inhibitoren, 10 % TNF-Inhibitoren und 6 % andere zielgerichtete Therapien. Nach 3 Monaten lag die Gesamtansprechrate bei 24 % mit JAKi, 32 % mit Anti-IL‑6, 9 % mit Anti-IL‑1 und 0 % mit TNF-Inhibitoren oder anderen zielgerichteten Therapien, wobei hier die Therapiepersistenz bei den JAKi am besten war.

Ruxolitinib (Handelsname *Jakavi*), ein JAK-1/2-Hemmer, der zur Behandlung der Myelofibrose zugelassen ist (Startdosis 2‑mal 15 mg/Tag), war in der französischen Analyse der am häufigsten verwendete JAKi (87 % der mit JAKi behandelten Fälle) und erwies sich in dieser und anderen Analysen [4] als wirksamer als die weiteren JAKi (komplette Remission [CR] nach 3 Monaten 83 % gegenüber 18 %; nach 6 Monaten 87 % gegenüber 11 %).

Zusammenfassend lässt sich festhalten, dass JAKi (hier insbesondere Ruxolitinib) und IL-6i den anderen zielgerichteten Therapien für VEXAS überlegen zu sein scheinen, wobei auch hierunter des Öfteren keine anhaltende suffiziente Krankheitskontrolle zu erzielen ist.

Bezüglich einer zielgerichteten Therapie des mutierten UBA1-Klons gibt es aktuell 2 Therapieoptionen:

Azacitidin (Handelsname *Vidaza*) ist ein Cytidinanalogon, welches als hypomethylierender Wirkstoff für die Behandlung der CMML, des MDS sowie als Erhaltungstherapie bei AML zugelassen ist. Das Medikament wird subkutan in einer Dosierung von 75 mg/m^2^ täglich über 7 Tage injiziert, gefolgt von 21 Tagen Behandlungspause (28 Tage = Behandlungszyklus), über mindestens 6 Behandlungszyklen.

In einem Review von 2023 [5] konnte gezeigt werden, dass Azacitidin nach den JAKi die zweitbeste Therapieansprechrate (25 % „complete response“, 38,9 % „partial response“) aufwies. Kürzlich konnte bei 4 Patienten mit VEXAS und MDS in einer Langzeitbeobachtung ebenfalls ein gutes Therapieansprechen nachgewiesen werden, wobei die UBA1-mutierten Klone auf sehr niedrige oder sogar nicht nachweisbare Werte reduziert werden konnten, was ebenfalls eine deutliche Reduzierung der Glukokortikoiddosis ermöglichte [6].

Ein potenziell kurativer Behandlungsansatz ist die allogene Stammzelltransplantation (alloHSCT), die beim Hochrisiko-MDS bereits eine Therapieoption darstellt. Die Datenlage hierzu ist jedoch noch sehr heterogen, da keine etablierten Studienprotokolle für das VEXAS-Syndrom existieren und die Beobachtungszeiträume teilweise stark variieren [7]: Je nach Publikation liegt die transplantationsbedingte Sterblichkeit bei 0–25,8 %. Aktuell sollte diese Therapieoption nur in enger hämatoonkologischer Absprache und nur für eher „jüngere und fittere Patienten“ im Einzelfall in Betracht gezogen werden.

Bezüglich der krankheitsassoziierten Komorbiditäten ist hervorzuheben, dass VEXAS-Patienten ein erhöhtes Infektionsrisiko haben und dieses bei der Erkrankung zu einem deutlich erhöhten Mortalitätsrisiko beiträgt. Daten aus der French VEXAS Group konnten in einer Kohorte von 74 Patienten insgesamt 133 schwere Infektionen nachweisen (davon 59 % pulmonale Infektionen, 10 % Hautinfektionen und 9 % Infektionen des Urogenitaltraktes) [7]. Neben SARS-CoV‑2 (28 %) und Pneumocystis jirovecii (PJP) (19 %) sah man in der Kohorte interessanterweise 21 % Legionelleninfektionen sowie 11 % invasive Pilzinfektionen.

Eine weitere aktuelle Arbeit aus den USA screente 94 VEXAS-Patienten bezüglich opportunistischer Infektionen [9]: Davon wurde bei 6 % eine PJP nachgewiesen, 15 % hatten eine Reaktivierung von Alpha-Herpesviren (hauptsächlich Varizella-Zoster-Virus [VZV]), und bei 10 % wurden nichttuberkulöse Mykobakterien (NTM) nachgewiesen. Eine Infektion mit PJP und NTM erhöhte das Sterberisiko signifikant, ebenso eine frühere Herpes-simplex-Virus(HSV)-Reaktivierung. Die Prophylaxe von PJP und VZV reduzierte die Infektionsraten signifikant (NNT von 4 und 7), was die Bedeutung einer prophylaktischen antiinfektiven Behandlung der Patienten unterstreicht.

Weiterhin konnte für VEXAS gezeigt werden, dass ein deutlich erhöhtes Risiko für thromboembolische Komplikationen besteht. Eine Auswertung von 119 Patienten in den USA ergab Thrombosen bei 49 % der Patienten, davon 41 % venöse (VTE). Fast zwei Drittel der VTE waren unprovoziert, 41 % waren rezidivierend, und 20 % traten trotz Antikoagulation auf. Die kumulative Inzidenz von VTE betrug 17 % im ersten Erkrankungsjahr und 40 % nach 5 Jahren. Weiterhin wurden in der Kohorte auch arterielle Thrombosen bei 6 % nach 1 Jahr und 11 % nach 5 Jahren detektiert. Interessanterweise legen erste Daten nahe, dass das Thromboserisiko durch die Inflammation der Gefäßwand getriggert sein könnte (ähnlich wie beim Behçet-Syndrom), was darauf hinweist, dass hier wiederum die Kontrolle der systemischen Inflammation primär im Therapiefokus stehen sollte. Insbesondere beim Einsatz von JAKi konnte ein erhöhtes Risiko für das Auftreten von thromboembolischen Komplikationen bei VEXAS-Patienten gezeigt werden [5]. Auch wenn aktuell keine allgemeine Empfehlung für eine prophylaktische Antikoagulation gegeben werden kann, empfehlen sich eine engmaschige Evaluation des allgemeinen Thromboserisikos der Patienten sowie eine liberale Thromboseprophylaxe in Risikosituationen (z. B. Hospitalisierung oder Einsatz von JAKi). In Anlehnung an onkologische Empfehlungen würde ich hier sowohl NMH oder NOAK präferieren.

In Abb. 2 ist mein Vorschlag für den Behandlungsalgorithmus des VEXAS-Syndroms, adaptiert nach Heiblig et al., [8] aufgeführt.

**Abb. 2:**
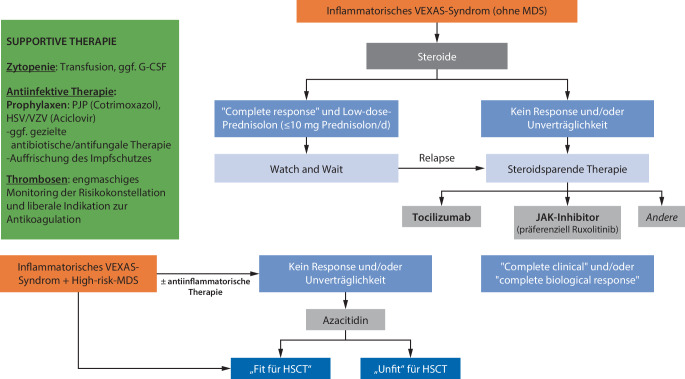
VEXAS-Syndrom Therapiealgorithmus. (Mod. nach Heiblig et al. [8])

Da sich im Laufe der Erkrankung bei bis zu einem Drittel der Betroffenen eine Transfusionsbedürftigkeit einstellt [10], sind eine engmaschige Monitorierung sowie eine zusätzliche hämatologische Anbindung (auch im Hinblick auf die Evaluation einer Therapie mit Azacitidin oder alloHSCT sowie zur Beurteilung des Vorliegens und des Schweregrades eines MDS) sinnvoll.

Abschließend lässt sich festhalten, dass die Behandlung des VEXAS-Syndroms eine interdisziplinäre Herausforderung darstellt. Die Erkrankung ist mit einer deutlich erhöhten Morbidität und Mortalität verbunden. Dies sollte in der Kommunikation mit den Betroffenen klar adressiert werden, und Therapieziele (ggf. auch Limitationen) insbesondere im Hinblick auf die Lebensqualität und die weitere Lebenserwartung sollten gemeinsam abgestimmt werden.

## Fazit

- Nach aktuellem Kenntnisstand (08/2024) sind JAK-Inhibitoren und IL-6-Inhibitoren die am besten wirksamen antiinflammatorischen Medikamente zur Behandlung des VEXAS-Syndroms, wobei auch hierunter des Öfteren keine anhaltende Krankheitskontrolle zu erzielen ist.
- Ruxolitinib scheint den anderen JAK-Inhibitoren überlegen zu sein.
- Bei unzureichender antiinflammatorischer Kontrolle kann der Einsatz von Azacitidin in Erwägung gezogen werden.
- Für jüngere und fittere Patienten kann im Einzelfall eine allogene Stammzelltransplantation mit kurativem Behandlungsansatz in Erwägung gezogen werden.
- Aufgrund des erhöhten Infektrisikos ist eine dauerhafte PJP- und HSV/VZV-Prophylaxe (mit Cotrimoxazol [3-mal 960 mg p.o/Woche] und Aciclovir [400 mg 1‑0-1]) zu empfehlen.
- Aufgrund des deutlich erhöhten Thromboserisikos sollte engmaschig das individuelle Patientenrisiko evaluiert werden und niederschwellig eine Antikoagulation erfolgen.
- Eine hämatoonkologische Mitbetreuung der Patienten ist sinnvoll.
